# Characterization of Chemical Composition of Pericarpium Citri Reticulatae Volatile Oil by Comprehensive Two-Dimensional Gas Chromatography with High-Resolution Time-of-Flight Mass Spectrometry

**DOI:** 10.1155/2013/237541

**Published:** 2013-04-28

**Authors:** Kunming Qin, Lijuan Zheng, Hao Cai, Gang Cao, Yajing Lou, Tulin Lu, Yachun Shu, Wei Zhou, Baochang Cai

**Affiliations:** ^1^Engineering Center of Ministry of Education for Standardization of Chinese Medicine Processing, Nanjing University of Chinese Medicine, Nanjing 210023, China; ^2^Nanjing Haichang Chinese Medicine Group Co., Ltd., Nanjing 210061, China; ^3^Research Center of TCM Processing Technology, Zhejiang Chinese Medical University, Hangzhou 310053, China; ^4^Affiliated Hospital of Nanjing University of Chinese Medicine, Nanjing 210029, China

## Abstract

Pericarpium Citri Reticulatae (Chenpi in Chinese) has been widely used as an herbal medicine in Korea, China, and Japan. Chenpi extracts are used to treat indigestion and inflammatory syndromes of the respiratory tract such as bronchitis and asthma. This thesis will analyze chemical compositions of Chenpi volatile oil, which was performed by comprehensive two-dimensional gas chromatography with high-resolution time-of-flight mass spectrometry (GC × GC-HR-TOFMS). One hundred and sixty-seven components were tentatively identified, and terpene compounds are the main components of Chenpi volatile oil, a significant larger number than in previous studies. The majority of the eluted compounds, which were identified, were well separated as a result of high-resolution capability of the GC × GC method, which significantly reduces, the coelution. **β**-Elemene is tentatively qualified by means of GC × GC in tandem with high-resolution TOFMS detection, which plays an important role in enhancing the effects of many anticancer drugs and in reducing the side effects of chemotherapy. This study suggests that GC × GC-HR-TOFMS is suitable for routine characterization of chemical composition of volatile oil in herbal medicines.

## 1. Introduction

Pericarpium Citri Reticulatae (Chenpi in Chinese) has been widely used as an herbal medicine for a long time in China, Korea, and Japan, for its pharmacologic activity, rich resources, low toxicity, and costs. Chenpi is the dried ripe fruit peel of *Citrus reticulata* Blanco and its cultivars, gathered from September to December [1]. Their main cultivars are *Citrus reticulata* “Chachi,” Citrus Reticulata “Dahongpao,” and *Citrus erythrosa* Tanaka. In Chinese people's traditional use, Chenpi is mostly utilized to eliminate phlegm and strengthen spleen [2]. Moreover, Chenpi is extensively added to food as a condiment. 

It is well known that Chenpi contains various bioactive compounds, such as flavonoids, phenolic acids, and limonoids [2, 3]. In the present study, most reports on Chenpi focus on phenolic compounds and flavonoids [3–7], but few focus on volatile compounds which also have strong pharmacologic bioactivities. For example, high-performance liquid chromatography (HPLC), high-speed countercurrent chromatography (HSCCC), and capillary electrophoresis (CE) have been applied for the determination of phenolic compounds and flavonoids of Chenpi [8–10]. However, reviewing the literature, it seems that the chemical composition of the volatile oil of Chenpi has been little investigated [11]. Furthermore, the volatile compounds of Chenpi may contribute to pharmacological effects of Chenpi extracts reported above [12, 13]. Therefore, a method able to rapidly identify the volatile compounds of Chenpi could be a useful tool for the purpose of a complete phytochemical analysis.

**Figure 1:**
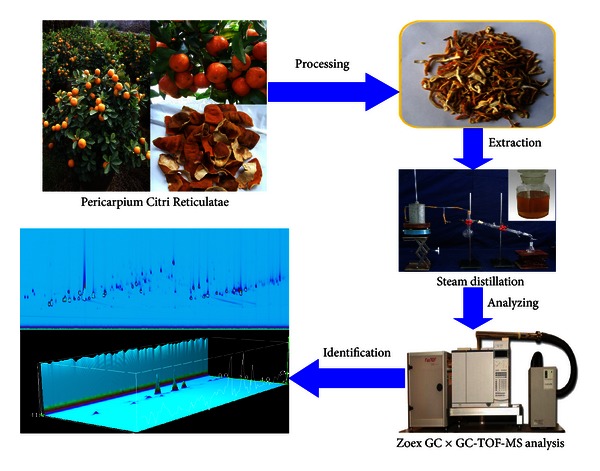
Flow chart of the chemical composition study of Chenpi volatile oil by GC × GC-HR-TOFMS.

Gas chromatography-mass spectroscopy (GC-MS) has been used for the qualitative analysis of the volatile constituents in Chenpi [11]. But it is difficult to achieve the complete separation of minor volatile components and many coelution volatile constituents. To solve these problems, it is necessary to use multidimensional gas chromatography. Comprehensive two-dimensional gas chromatography with high-resolution time-of-flight mass spectrometry (GC × GC-HR-TOFMS) is a new developed powerful and versatile analytical tool, which combines two powerful analytical technologies with complementary attributes [14, 15]. GC × GC separates chemical species with two capillary columns interfaced by a modulator that traps and concentrates eluents from the first column, and it then introduces them into the second column, producing a full secondary chromatogram for each single data point of a traditional one-dimensional separation [16, 17]. HR-TOFMS provides mass precision that is fine enough to distinguish elemental compositions, providing a more definitive basis for molecular identification. GC × GC is important for HR-TOFMS because the better separations significantly reduce the coelution and the problems of mass spectral mixing. And, HR-TOFMS is important for GC × GC because the structural and compositional information available with HR-TOFMS aids in the interpretation of the rich, complex data from GC × GC separations [18]. GC × GC-TOFMS has been successfully applied in the volatile oil study and greatly improves the result of component separation and identification [19, 20]. In this study, the volatile oil of Chenpi was firstly separated and detected with GC × GC-HR-TOFMS (Figure 1).

## 2. Materials and Methods

### 2.1. Samples

Chenpi sample (fruit peels of *Citrus reticulate* “Dahongpao”) was collected from Zigong in Sichuan province, China. The sample was authenticated by Professor Chen Jianwei from Nanjing University of Traditional Chinese Medicine, China.

### 2.2. Extraction of Volatile Oil

After the sample was dried for 2 h at 45°C and smashed, 50 g of sample was swollen with 600 mL of distilled water in a standard extractor for extracting volatile oil for 3 h. Then, the volatile oil was dried over anhydrous sodium sulphate until all the water was dried and then stored in the dark glass bottle at 4°C prior to GC × GC-HR-TOFMS analysis.

### 2.3. GC-MS System and GC × GC-HR-TOFMS Apparatus

GC × GC separations were performed by Tofwerk AG (Thun, Switzerland) on an Agilent 7890 A GC and 7693 autosampler with: 1 *μ*L splitless injection; column one DB-XLB (Agilent), 15 m × 0.25 mm, 0.25 *μ*m film thickness; column two BPX-50 (SGE), 1 m × 0.1 mm, 0.1 *μ*m film thickness; oven temperature from 50 to 230°C at 2.0°C min^−1^ ramp; inlet pressure from 35 PSI to 61.5 PSI at 0.28 PSI min^−1^; injection temperature 250°C; transfer line temperature 300°C; Zoex ZX2 thermal modulator with a 7 s modulation period, 300 ms modulation duration, 375°C hot jet temperature, 18 L min^−1^ cold jet nitrogen flow rate, and 40 PSI hot jet nitrogen pressure. The Zoex FasTOF time-of-flight- (TOF-) HRMS system used 70 eV EI ion source, 280°C ion source temperature, a mass range of *m/z* 50–450 with 4000 FWHM resolution, and 100 spectra per second acquisition rate.

### 2.4. Data Conversion and Peak Table Generation

The final data for each chromatogram is an array of 1000 × 600 data points, each data point with a HRMS vector of 40 K intensities. Thus, each chromatogram has 24 billion values requiring 96 gigabytes for representing for single-precision floating point numbers without compression. The set of 18 chromatograms has more than 1.7 terabytes of uncompressed data. The data were compressed and stored by the Zoex FasTOF system to HDF5-format files and were processed with GC Image GC × GC Software R2.1. In order to manage such large files on computers with limited random access memory (RAM), GC Image Software maintains a chromatogram with integer mass or centroid-resampled spectra in RAM and accesses the HR-MS data from disk as needed. GC Image can export raw data and computed results to nonproprietary file formats for processing with external software. The components can be quantified by Zoex software (Zoex Corp, Lincoln, NE, USA).

All peaks with signal-to-noise ratio higher than 100 were found in the raw GC × GC chromatogram. The workstation can automatically give the parameters such as similarity, reverse, and probability of peaks via comparing them with the compounds in the library. The results were combined in a peak table. The NIST/EPA/NIH Mass Spectral Library Version 2.0 was used in this work.

## 3. Results and Discussion

### 3.1. Qualitative Analysis of Chenpi Volatile Oil

**Figure 2:**
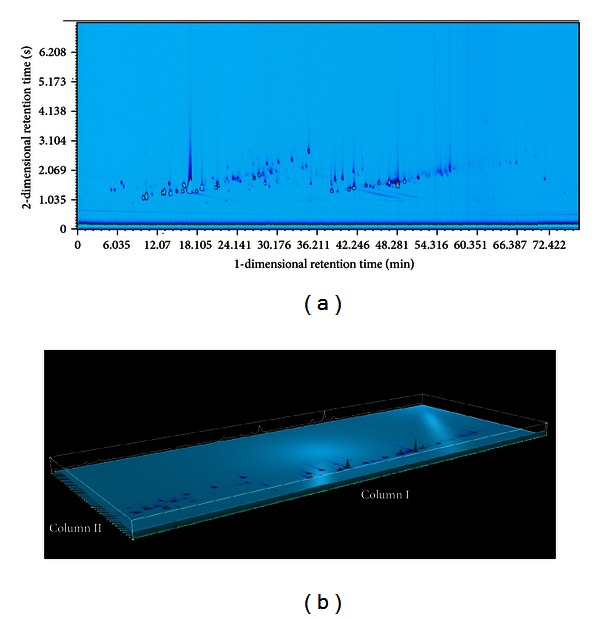
GC × GC-HR-TOFMS chromatogram (a) and three-dimensional chromatogram (b) of Chenpi volatile oil.

The column system is nearly orthogonal and provides a structured separation. A typical two-dimensional separation/total ion chromatogram (TIC) and three-dimensional chromatogram are shown in Figure 2. In the GC × GC system, compounds are separated by volatility difference on the first dimension nonpolar column and by polarity on the second medium-polar column. The GC × GC system accomplishes the true orthogonal separation on account for both the change of the polarity of two fixed phases and the linear temperature programming.

Using GC × GC-HR-TOFMS, the quantity of the detected components was up to 834. Compared to the traditional identification method such as GC-MS, the analysis from GC × GC-HR-TOFMS becomes more reliable, relying on the combined identification information including retention times, similarity, reverse match factor, and probability. The similarity and reverse match factors indicate how well a mass spectrum matches the library spectrum, but the isomers have similar mass spectra. In this case, the probability is used to determine whether the peaks with the same name belong to one compound or several compounds. The GC × GC-HR-TOF/MS software was used to find all the peaks in the raw GC × GC chromatogram. A library search was carried out for all the peaks using the NIST/EPA/NIH version 2.0, and the results were combined in a single peak table. A similarity and reverse match factor above 583 and 612, respectively, indicates that an acquired mass spectrum usually shows a good match with the library spectrum. Because of the numerous isomers present in volatile oils, especially within monoterpenes and sesquiterpenes, more attention should be paid for identification using mass spectra. In order to enhance the reliability of the identification by MS, both similarity and reverse match factor should be used. According to our experience and the literature data [18–20], 167 compounds with good match were tentatively identified including 50 monoterpenes, 36 sesquiterpenes, 31 esters and acids, 9 aldehydes and ketones, 6 alcohols, 3 ethers, 12 phenyl compounds, and 20 other components. Compounds have lower search probabilities than these counted as unknowns, and were disqualified for Kovats index comparison.  Table 1 listed 167 components identified in Chenpi volatile oil. The volatile fraction is characterized by high percentages of monoterpenes, sesquiterpenes, and esters, including *β*-elemene, p-mentha-1(7),8(10)-dien-9-ol, and limonene. In this study, many components have also been tentatively identified, which were found in Chenpi volatile oil for the first time such as globulol and isoledene. There is high possibility that they will be literally useful for further pharmaceutical research of Chenpi volatile oil.

### 3.2. Group Separation of Chenpi Volatile Components

**Figure 3:**
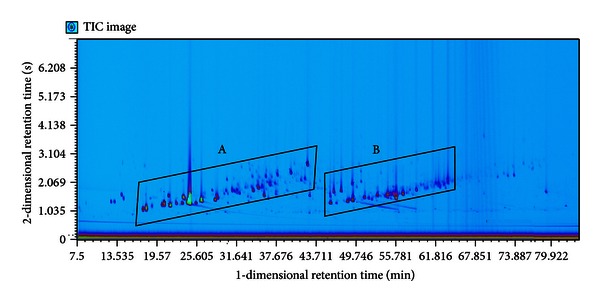
The GC × GC contour plot of Chenpi volatile oil group separation result. Regions marked by squares (A) and (B) were identified mainly as monoterpenes and sesquiterpenes, respectively.

In GC × GC-HR-TOFMS analysis, the 167 identified volatile components in Chenpi volatile oil were mainly classified into two groups that can be seen in Figure 3. Based on GC × GC-HR-TOFMS, it can be found that the peaks in areas A and B are monoterpenes and sesquiterpenes, respectively. These monoterpenes and sesquiterpenes are mainly alkenes, alcohols, and ethers. It was also found that a lot of saturated and unsaturated fatty acid esters and phenyl compounds constitute the Chenpi volatile oil. This study demonstrates that GC × GC-HR-TOFMS is a powerful separation and identification tool that allows for the identification and group separation of a much larger number of complex volatile oil components.

### 3.3. Identification of Three Coelution Volatile Components in Chenpi Volatile Oil

**Figure 4:**
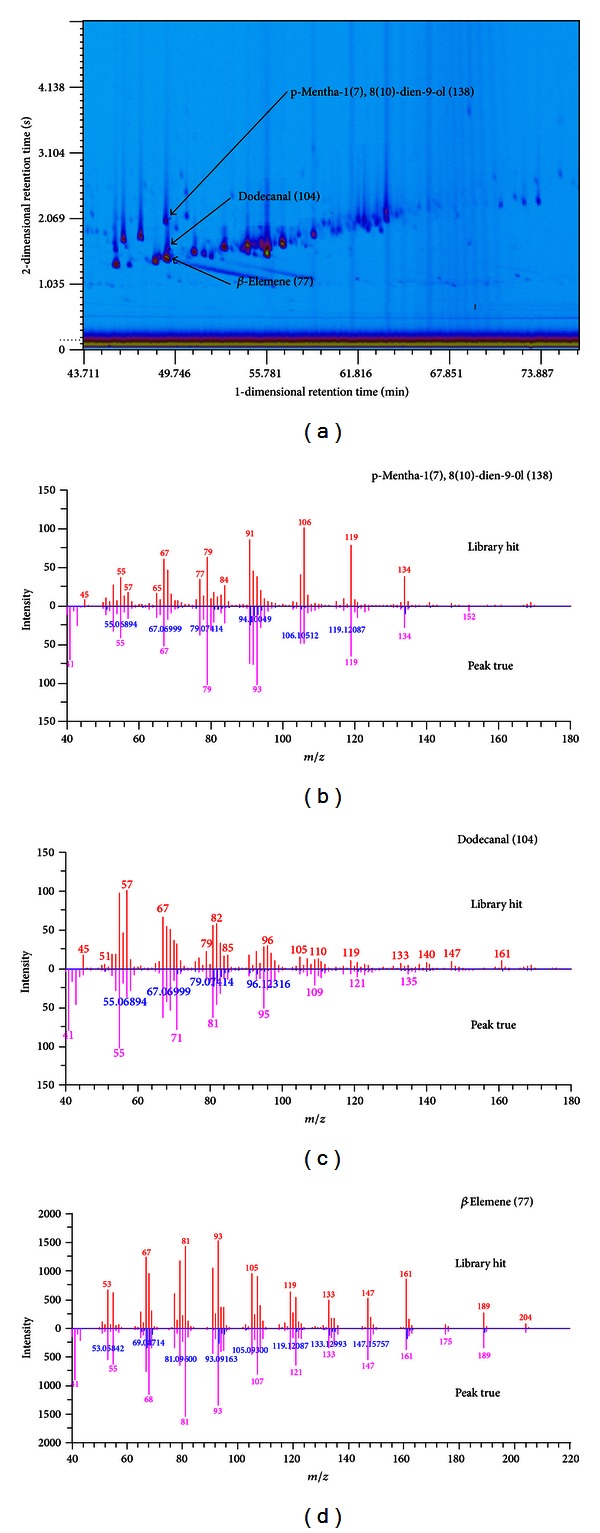
Details of three coelution volatile components (peak 77, 104, 138) in GC × GC chromatogram. The spectra of *β*-elemene (peak 77), dodecanal (peak 104), and p-mentha-1(7),8(10)-dien-9-ol (peak 138) in sample and in NIST library, respectively.

The high-resolution mass spectra in the TIC can be used for accurate identification of volatile compounds in Chenpi volatile oil, and these identified compounds will be significant to the further pharmaceutical research. For example, Figure 4 compares the high resolution mass spectrum of the blob (peak) marked with 138 (49.14 min, 2.02 s), 104 (49.26 min, 1.58 s), and 77 (49.14 min, 1.45 s), head-to-tail with the mass spectrum of p-mentha-1(7),8(10)-dien-9-ol, dodecanal, and *β*-elemene TMS from the NIST/EPA/NIH library mass spectra. For p-mentha-1(7),8(10)-dien-9-ol, the forward match factor is 806; reverse match factor is 855; and probability is 20.12%. For dodecanal, the forward match factor is 763; reverse match factor is 773; and probability is 7.16%. For *β*-elemene, the forward match factor is 911; reverse match factor is 914; and probability is 17.11%. The above three volatile components cannot be clearly separated or identified by traditional one-dimensional gas chromatography or GC-MS method, because they are coelution volatile components, which have very similar chemical properties including volatility and polarity. In this study, the three coelution volatile components in Chenpi volatile oil were well separated and identified by GC × GC-HR-TOFMS, which have not been reported in other studies (Figure 4).

This study showed that GC × GC-HR-TOFMS represents a powerful separation and analysis tool for the analysis of complex volatile oils of herbal medicines. GC × GC-HR-TOFMS can give the information about the formula and structures, can provide the opportunity for differentiating different volatile oils, can give the subtle differences of the oils from different areas, and can find new compounds that have the possible pharmaceutical effect on some diseases. 

## 4. Conclusions

In this study, GC × GC-HR-TOFMS not only tentatively identified 167 volatile components in Chenpi volatile oil, but also provided several kinds of identification information that make the result more reliable. Among 167 components, there are 50 monoterpenes, 36 sesquiterpenes, 31 esters and acids, 9 aldehydes and ketones, 6 alcohols, 3 ethers, 12 phenyl compounds, and 20 other components. Monoterpenes and sesquiterpenes are the main components of Chenpi volatile oil. This study demonstrates a dependable method for the qualitative analysis of volatiles, which can achieve an accurate and comprehensive chromatographic profile with a low contamination risk and cost, as well as shortened sample preparation time. GC × GC-HR-TOFMS will play an important role in the analysis of volatile oils of herbal medicines in the future.

## Figures and Tables

**Table 1:** 167 main volatile components identified in the Chenpi volatile oil.

No.	Compound name	Peak I/min	Peak II/s	Volume	Library formula	Library probability	Library CAS no.
(1)	Nonanal	28.62	1.6	2367.153	C_9_H_18_O	50.1	124-19-6
(2)	Pyrrolizidine-3-one-5-ol, ethyl ether	53.49	2.74	53.1709	C_9_H_15_NO_2_	17.7	0-00-0
(3)	2-Methoxy-4-vinylphenol	42.38	2.73	2959.926	C_9_H_10_O_2_	59.39	7786-61-0
(4)	Styrene	14.14	1.65	731.272	C_8_H_8_	36.4	100-42-5
(5)	Octanal	21.38	1.58	2235.185	C_8_H_16_O	62.19	124-13-0
(6)	Ethylbenzene	12.57	1.39	484.4996	C_8_H_10_	64.86	100-41-4
(7)	Pterin-6-carboxylic acid	11.24	0.82	50.3913	C_7_H_5_N_5_O_3_	40.94	948-60-7
(8)	Hexadecane, 1,1-bis(dodecyloxy)-	77.27	1.2	102.3988	C_40_H_82_O_2_	9.24	56554-64-4
(9)	1-Heptatriacotanol	75.09	2.76	169.2274	C_37_H_76_O	54.56	105794-58-9
(10)	Cholestan-3-ol, 2-methylene-, (3*β*,5*α*)-	64.35	2.11	268.4351	C_28_H_48_O	13.09	22599-96-8
(11)	[5,9-Dimethyl-1-(3-phenyl-oxiran-2-yl)-deca-4,8-dienylidene]-(2-phenyl-aziridin-1-yl)-amine	66.4	2.23	151.311	C_28_H_34_N_2_O	44.04	0-00-0
(12)	1,1′-(4-Methyl-1,3-phenylene)bis[3-(5-benzyl-1,3,4-thiadiazol-2-yl)urea]	23.55	2.15	106.0574	C_27_H_24_N_8_O_2_S_2_	24.95	0-00-0
(13)	Morphinan-4,5-epoxy-3,6-di-ol, 6-[7-nitrobenzofurazan-4-yl]amino-	46.49	0.79	105.5334	C_26_H_27_N_5_O_6_	9.48	0-00-0
(14)	Benzene, 1,1′-[3-(3-cyclopentylpropyl)-1,5-pentanediyl]bis-	7.74	1.18	149.2865	C_25_H_34_	15.18	55191-62-3
(15)	2-(2-Azepan-1-yl-2-oxoethyl)-1-hydroxy-1-phenyl-octahydro-pyrido[1,2-a]azepin-4-one	76.42	2.77	142.1083	C_24_H_34_N_2_O_3_	52.15	0-00-0
(16)	6,9,12,15-Docosatetraenoic acid, methyl ester	59.4	1.77	55.943	C_23_H_38_O_2_	19.28	17364-34-0
(17)	2-[4-Methyl-6-(2,6,6-trimethylcyclohex-1-enyl)hexa-1,3,5-trienyl]cyclohex-1-en-1-carboxaldehyde	53.49	2.15	64.4497	C_23_H_32_O	25.57	0-00-0
(18)	Naphthalen-2-yl-acetic acid, 6-hydroxy-6-methyl-cyclodecyl ester	41.54	2.63	65.5915	C_23_H_30_O_3_	27.3	0-00-0
(19)	Z-5-Methyl-6-heneicosen-11-one	80.89	1.11	115.1736	C_22_H_42_O	8.27	0-00-0
(20)	2H-Pyran, 2-(7-heptadecynyloxy)tetrahydro-	61.45	1.91	133.6559	C_22_H_40_O_2_	14.4	56599-50-9
(21)	Doconexent	17.88	1.63	127.9331	C_22_H_32_O_2_	40.98	6217-54-5
(22)	9,12,15-Octadecatrienoic acid, 2,3-dihydroxypropyl ester, (Z,Z,Z)-	31.16	2.22	105.1463	C_21_H_36_O_4_	19.78	18465-99-1
(23)	8,11,14-Eicosatrienoic acid, methyl ester, (Z,Z,Z)-	42.75	2.02	216.8234	C_21_H_36_O_2_	8.42	21061-10-9
(24)	5,8,11-Eicosatrienoic acid, methyl ester	57.59	2.03	96.1455	C_21_H_30_O_2_	43.95	0-00-0
(25)	cis-5,8,11,14,17-Eicosapentaenoic acid	24.52	4.25	53.7632	C_20_H_30_O_2_	13.99	10417-94-4
(26)	5,8,11,14-Eicosatetraynoic acid	58.8	3.17	144.9613	C_20_H_24_O_2_	29.78	1191-85-1
(27)	1,16-Cyclocorynan-17-oic acid, 19,20-didehydro-, methyl ester, (16S,19E)-	12.21	0.64	88.5548	C_20_H_22_N_2_O_2_	53.45	6393-66-4
(28)	Octadecane, 6-methyl-	82.09	1.25	184.9235	C_19_H_40_	18.41	10544-96-4
(29)	2-Methyl-E,E-3,13-octadecadien-1-ol	64.83	2.01	124.0941	C_19_H_36_O	9.74	0-00-0
(30)	Z,Z,Z-4,6,9-Nonadecatriene	26.81	1.7	109.996	C_19_H_34_	21.05	0-00-0
(31)	6,9,12-Octadecatrienoic acid, methyl ester	55.78	2.92	63.7153	C_19_H_32_O_2_	28.52	2676-41-7
(32)	Z,Z,Z-1,4,6,9-Nonadecatetraene	21.5	1.8	65.4446	C_19_H_32_	14.64	0-00-0
(33)	12,15-Octadecadienoic acid, methyl ester	49.14	2.35	158.5734	C_19_H_30_O_2_	19.33	57156-95-3
(34)	10,13-Octadecadienoic acid, methyl ester	50.47	2.47	161.0184	C_19_H_30_O_2_	15.45	18202-24-9
(35)	2,5-Octadecadienoic acid, methyl ester	59.52	1.99	254.7258	C_19_H_30_O_2_	10.15	57156-91-9
(36)	2,2,4,4-Tetramethyl-6-(1-oxo-3-phenylprop-2-enyl)-cyclohexane-1,3,5-trione	70.27	0.49	83.2401	C_19_H_20_O_4_	21.98	0-00-0
(37)	Gentamicina	63.63	3.21	62.6103	C_18_H_36_N_4_O_10_	27.05	13291-74-2
(38)	Z-10-Methyl-11-tetradecen-1-ol propionate	40.33	2	70.3719	C_18_H_34_O_2_	10.53	0-00-0
(39)	10-Heptadecen-8-ynoic acid, methyl ester, (E)-	34.18	2.06	63.2456	C_18_H_30_O_2_	14.6	16714-85-5
(40)	*α*-L-Fucopyranose 1,2:3,4-bis(benzeneboronate)	67.13	2.28	180.5156	C_18_H_18_B_2_O_5_	25.89	102281-26-5
(41)	3-(O-Anisidinomethyl)-5-(3-fluorobenzylidene)-2,4-thiazolidinedione	34.66	2.46	82.9072	C_18_H_15_FN_2_O_3_S	9.43	302954-96-7
(42)	1-Hexadecanol, 2-methyl-	44.19	1.03	65.1528	C_17_H_36_O	11.82	2490-48-4
(43)	10-Methyl-E-11-tridecen-1-ol propionate	33.33	1.56	164.2353	C_17_H_32_O_2_	6.69	0-00-0
(44)	13-Heptadecyn-1-ol	40.09	2.25	78.5129	C_17_H_32_O	12.04	56554-77-9
(45)	4,7,10-Hexadecatrienoic acid, methyl ester	32.97	2.05	83.5894	C_17_H_28_O_2_	7.98	17364-31-7
(46)	Methyl 5,7-hexadecadienoate	53.61	1.7	58.765	C_17_H_26_O_2_	14.34	0-00-0
(47)	3-(5-Benzyloxy-3-methylpent-3-enyl)-2,2-dimethyloxirane	35.02	2.61	96.359	C_17_H_24_O_2_	8.95	0-00-0
(48)	Falcarinol	37.07	1.57	55.0178	C_17_H_24_O	35.51	21852-80-2
(49)	tert-Hexadecanethiol	70.27	1.03	151.5898	C_16_H_34_S	13.87	25360-09-2
(50)	n-Hexadecanoic acid	79.08	1.74	705.7247	C_16_H_32_O_2_	43.05	10-3-1957
(51)	Cyclopentaneundecanoic acid	46.49	1.62	94.519	C_16_H_30_O_2_	13.97	6053-49-2
(52)	9-Hexadecenoic acid	65.56	1.91	89.144	C_16_H_30_O_2_	13.29	2091-29-4
(53)	Formic acid, 3,7,11-trimethyl-1,6,10-dodecatrien-3-yl ester	51.92	1.71	99.1647	C_16_H_26_O_2_	23.72	0-00-0
(54)	1,3-Dioxolane, 2-heptyl-4-phenyl-	7.74	0.67	137.1547	C_16_H_24_O_2_	20.64	55668-40-1
(55)	Peyonine	24.64	0.58	54.9972	C_16_H_19_NO_5_	27.28	19717-25-0
(56)	12,14,14-Trimethyl-3,6,9-trioxapentadecan-1-ol	66.52	1.18	58.5099	C_15_H_32_O_4_	16.94	55489-54-8
(57)	Isocalamendiol	73.65	2.76	52.3348	C_15_H_26_O_2_	24.27	0-00-0
(58)	Geranyl isovalerate	53.37	1.03	77.0183	C_15_H_26_O_2_	11.72	109-20-6
(59)	Cubeduel	63.26	1.88	203.2965	C_15_H_26_O	24.8	0-00-0
(60)	*α*-Cadinol	63.63	2.07	649.3081	C_15_H_26_O	24.36	481-34-5
(61)	Cubenol	62.42	1.9	408.4446	C_15_H_26_O	16.17	21284-22-0
(62)	.tau.-Cadinol	63.02	2	707.983	C_15_H_26_O	11.7	11-1-5937
(63)	*α*-Acorenol	63.63	2.17	1949.884	C_15_H_26_O	9.72	0-00-0
(64)	Globulol	60.13	1.88	278.3377	C_15_H_26_O	6.4	51371-47-2
(65)	7-Epi-cis-sesquisabinene hydrate	54.57	1.3	81.2625	C_15_H_26_O	6.4	0-00-0
(66)	Limonen-6-ol, pivalate	44.56	1.85	78.8648	C_15_H_24_O_2_	24.04	0-00-0
(67)	Aromadendrene oxide-(2)	62.78	2.09	96.7312	C_15_H_24_O	15.84	0-00-0
(68)	Caryophyllene oxide	59.88	2	161.5517	C_15_H_24_O	13.15	1139-30-6
(69)	Spiro[4.5]dec-6-en-8-one, 1,7-dimethyl-4-(1-methylethyl)-	38.16	2.27	73.2272	C_15_H_24_O	7.02	39510-36-6
(70)	Humulene	53	1.61	5204.711	C_15_H_24_	44.49	6753-98-6
(71)	(Z,E)-*α*-farnesene	55.78	1.52	31067.6	C_15_H_24_	42.94	26560-14-5
(72)	*α*-Copaene	48.42	1.4	7508.707	C_15_H_24_	42.37	0-00-0
(73)	*δ*-Elemene	45.88	1.33	5692.139	C_15_H_24_	38.01	20307-84-0
(74)	Naphthalene, 1,2,3,5,6,8a-hexahydro- 4,7-dimethyl-1-(1-methylethyl)-, (1S-cis)-	56.75	1.68	12956.64	C_15_H_24_	31	483-76-1
(75)	1,3,6,10-Dodecatetraene, 3,7,11-trimethyl-, (Z,E)-	54.94	1.51	196.9983	C_15_H_24_	29.01	26560-14-5
(76)	Caryophyllene	50.95	1.54	3006.936	C_15_H_24_	17.9	87-44-5
(77)	*β*-Elemene	49.14	1.45	20347.87	C_15_H_24_	17.11	515-13-9
(78)	*α*-Guaiene	52.16	1.47	556.5085	C_15_H_24_	16.63	12-1-3691
(79)	Cyclohexane, 1-ethenyl-1-methyl-2,4-bis (1-methylethenyl)-	52.76	1.23	160.6527	C_15_H_24_	16.42	110823-68-2
(80)	1,6-Cyclodecadiene, 1-Methyl-5-methylene-8-(1-methylethyl)-, [S-(E,E)]-	54.45	1.65	14112	C_15_H_24_	15.25	23986-74-5
(81)	*γ*-Elemene	51.68	1.51	742.7373	C_15_H_24_	15.14	29873-99-2
(82)	Naphthalene, 1,2,3,4,4a,7- hexahydro-1,6-dimethyl-4-(1-methylethyl)-	57.35	1.71	200.7951	C_15_H_24_	14.55	16728-99-7
(83)	Cyclohexane, 1-ethenyl-1-methyl- 2,4-bis(1-methylethenyl)-, [1S-(1*α*,2*β*,4*β*)]-	48.66	1.47	549.6758	C_15_H_24_	10.66	515-13-9
(84)	*β*-Copaene	54.45	1.55	561.4817	C_15_H_24_	10.53	0-00-0
(85)	*γ*-Elemene	58.8	1.82	1698.139	C_15_H_24_	8.31	29873-99-2
(86)	*β*-Guaiene	57.59	1.75	60.1417	C_15_H_24_	7.16	88-84-6
(87)	Guaia-1(10),11-diene	54.82	1.67	1093.799	C_15_H_24_	6.6	0-00-0
(88)	Isoledene	54.21	1.58	405.0377	C_15_H_24_	6.27	0-00-0
(89)	4,5-Di-epi-aristolochene	47.81	1.43	90.0937	C_15_H_24_	4.2	0-00-0
(90)	trans-calamenene	56.51	1.85	84.299	C_15_H_22_	38.35	0-00-0
(91)	*β*-Vatirenene	73.65	2.34	437.4147	C_15_H_22_	25.71	0-00-0
(92)	4,4-Dimethyl-3-(3-methylbut-3-enylidene)-2-methylenebicyclo[4.1.0]heptane	72.68	2.32	284.3733	C_15_H_22_	11.28	79718-83-5
(93)	7-Hydroxy-6,9a-dimethyl-3-methylene-decahydro-azuleno[4,5-b]furan-2,9-dione	75.21	3.61	61.6952	C_15_H_20_O_4_	11.61	0-00-0
(94)	Propanoic acid, 3-(2,3,6-trimethyl- 1,4-dioxaspiro[4.4]non-7-yl)-, methyl ester	33.93	2.7	84.8793	C_14_H_24_O_4_	19.92	0-00-0
(95)	2,5-Furandione, 3-(2-decenyl)dihydro-	40.33	2.76	154.1135	C_14_H_22_O_3_	11.38	62568-81-4
(96)	trans-(2-Decenyl)succinic anhydride	72.08	1.6	67.8793	C_14_H_22_O_3_	10.39	81949-64-6
(97)	1,4-Benzenediol, 2,6-bis(1,1-dimethylethyl)-	77.51	0.5	70.9573	C_14_H_22_O_2_	7.13	2444-28-2
(98)	Tetraacetyl-d-xylonic nitrile	58.32	1.62	100.275	C_14_H_17_NO_9_	18.48	0-00-0
(99)	*α*-Ionol	30.19	2.18	277.9312	C_13_H_22_O	15.16	25312-34-9
(100)	1-(2-Acetoxyethyl)-3,6-diazahomoadamantan-9-one oxime	73.28	0.5	55.2116	C_13_H_21_N_3_O_3_	8.78	0-00-0
(101)	1b,5,5,6a-Tetramethyl-octahydro-1-oxa-cyclopropa[a]inden-6-one	33.21	1.89	138.6104	C_13_H_20_O_2_	6.45	0-00-0
(102)	Pyrimidin-2-one, 4-[N-methylureido]-1-[4-methylaminocarbonyloxymethyl	68.94	0.38	54.3663	C_13_H_19_N_5_O_5_	23.12	0-00-0
(103)	2H-Indeno[1,2-b]furan-2-one, 3,3a,4,5,6,7,8,8b-octahydro-8,8-dimethyl	55.3	1.93	203.2335	C_13_H_18_O_2_	28.93	0-00-0
(104)	Dodecanal	49.26	1.58	1282.104	C_12_H_24_O	6.6	112-54-9
(105)	2,6-Octadien-1-ol, 3,7-dimethyl-, acetate, (Z)-	46.37	1.74	3059.872	C_12_H_20_O_2_	37.64	141-12-8
(106)	Geranyl acetate	47.45	1.79	3772.587	C_12_H_20_O_2_	28.7	105-87-3
(107)	(R)-Lavandulyl acetate	46.37	2.25	81.7472	C_12_H_20_O_2_	14.19	0-00-0
(108)	Geranyl vinyl ether	36.83	1.77	76.0262	C_12_H_20_O	14.31	0-00-0
(109)	3′-Hydroxyquinalbarbitone	39.61	1.5	51.4505	C_12_H_18_N_2_O_4_	10.72	839-21-4
(110)	Non-1-yn-5-en-9-aldehyde, 4-carbethoxy-	44.31	2.11	115.1319	C_12_H_16_O_3_	17.87	0-00-0
(111)	Undecanal	42.75	1.59	704.5031	C_11_H_22_O	33.86	112-44-7
(112)	Cyclohexane, 2-ethenyl-1,1-dimethyl-3-methylene-	30.31	1.34	135.2002	C_11_H_18_	23.83	95452-08-7
(113)	Cyclohexene, 2-ethenyl-1,3,3-trimethyl-	42.02	2.22	591.2578	C_11_H_18_	14.36	5293-90-3
(114)	Acetic acid, octyl ester	36.47	1.49	323.6557	C_10_H_20_O_2_	17.84	112-14-1
(115)	Decanal	35.74	1.61	7031.098	C_10_H_20_O	61.62	112-31-2
(116)	Cephrol	37.55	1.66	328.2375	C_10_H_20_O	12.34	40607-48-5
(117)	Linalol	28.74	1.52	3645.373	C_10_H_18_O	81.59	78-70-6
(118)	*α*-Terpineol	34.9	1.89	7440.985	C_10_H_18_O	67.75	98-55-5
(119)	Terpinen-4-ol	34.18	1.79	3293.372	C_10_H_18_O	50.03	562-74-3
(120)	Citronellal	32.12	1.69	615.3934	C_10_H_18_O	42.59	106-23-0
(121)	2-Cyclohexen-1-ol, 1-methyl-4-(1-methylethyl)-, cis-	30.31	1.67	159.2231	C_10_H_18_O	39.11	29803-82-5
(122)	Cyclohexanol, 1-methyl-4-(1-methylethenyl)-, cis-	31.64	1.8	726.8632	C_10_H_18_O	10.59	7299-41-4
(123)	2-Cyclohexen-1-ol, 2-methyl-5-(1-methylethyl)-, (1S-cis)-	36.11	1.85	101.2405	C_10_H_18_O	10.53	536-30-1
(124)	exo-2,7,7-trimethylbicyclo[2.2.1]heptan-2-ol	32.97	1.89	169.9902	C_10_H_18_O	8.15	0-00-0
(125)	5-Hepten-1-ol, 2-ethenyl-6-methyl-	45.76	1.68	397.1537	C_10_H_18_O	7.75	18479-48-6
(126)	Bicyclo[4.1.0]heptane, 3,7,7-trimethyl-, [1S-(1*α*,3*β*,6*α*)]-	45.76	1.58	368.8305	C_10_H_18_	9.72	2778-68-9
(127)	Desulphosinigrin	66.64	1.06	73.3518	C_10_H_17_NO_6_S	8.92	5115-81-1
(128)	R-Limonene	39.37	2.73	97.0311	C_10_H_16_O_3_	40.37	0-00-0
(129)	Limonene oxide, trans-	31.28	1.78	570.8168	C_10_H_16_O	54.24	4959-35-7
(130)	Limonene oxide, cis-	30.92	1.78	1068.476	C_10_H_16_O	40.43	13837-75-7
(131)	trans-p-Mentha-2,8-dienol	30.07	1.78	448.7691	C_10_H_16_O	34.35	0-00-0
(132)	3-Cyclohexene-1-acetaldehyde, *α*,4-dimethyl-	36.35	2.09	181.7561	C_10_H_16_O	31.38	29548-14-9
(133)	cis-p-Mentha-1(7),8-dien-2-ol	37.43	2.05	398.5489	C_10_H_16_O	26.42	0-00-0
(134)	(Z)-Carveol	35.5	1.97	452.2402	C_10_H_16_O	25.48	1197-06-4
(135)	trans-Carveol	36.71	2	1591.657	C_10_H_16_O	22.66	1197-07-5
(136)	cis-p-Mentha-1(7),8-dien-2-ol	33.93	1.91	336.4337	C_10_H_16_O	21.5	0-00-0
(137)	1-Cyclohexene-1-methanol, 4-(1-methylethenyl)-	35.62	1.89	195.9113	C_10_H_16_O	20.76	536-59-4
(138)	p-Mentha-1(7),8(10)-dien-9-ol	49.14	2.02	1162.984	C_10_H_16_O	20.12	29548-13-8
(139)	cis-p-Mentha-2,8-dien-1-ol	43.47	1.91	99.0278	C_10_H_16_O	7.83	3886-78-0
(140)	2,6-Dimethyl-3,5,7-octatriene-2-ol, E,E-	31.88	1.98	187.0291	C_10_H_16_O	7.6	0-00-0
(141)	(S)-(-)-(4-Isopropenyl-1-cyclohexenyl)methanol	35.38	1.89	172.7702	C_10_H_16_O	5.86	18457-55-1
(142)	Bicyclo[3.1.0]hex-2-ene, 4-methyl-1-(1-methylethyl)-	17.4	1.11	7918.412	C_10_H_16_	65.04	28634-89-1
(143)	*β*-Pinene	20.66	1.31	19964.34	C_10_H_16_	40.8	127-91-3
(144)	*β*-Ocimene	25.48	1.34	4519.345	C_10_H_16_	37.56	13877-91-3
(145)	*α*-Phellandrene	22.47	1.32	2085.008	C_10_H_16_	33.42	99-83-2
(146)	*β*-Myrcene	21.5	1.27	69260.99	C_10_H_16_	31.3	123-35-3
(147)	*α*-Pinene	17.88	1.16	48376.96	C_10_H_16_	23.82	80-56-8
(148)	Camphene	18.85	1.22	406.4708	C_10_H_16_	19.93	79-92-5
(149)	*β*-Phellandrene	20.29	1.27	4173.575	C_10_H_16_	19.68	555-10-2
(150)	D-Limonene	24.52	1.69	1784450	C_10_H_16_	19.59	5989-27-5
(151)	*α*-Terpinene	23.43	1.33	4905.547	C_10_H_16_	17.58	99-86-5
(152)	Isoterpinene	28.38	1.46	14607.02	C_10_H_16_	16.01	586-62-9
(153)	*γ*-Terpinene	26.33	1.5	189808.1	C_10_H_16_	15.28	99-85-4
(154)	1,5,5-Trimethyl-6-methylene-cyclohexene	26.33	1.86	305.9981	C_10_H_16_	9.81	514-95-4
(155)	Cyclohexene, 1-methyl-4-(1-methylethenyl)-, (S)-	24.52	3.31	51.7426	C_10_H_16_	9.58	5989-54-8
(156)	*γ*-Pyronene	23.07	1.27	60.3834	C_10_H_16_	5.33	514-95-4
(157)	5-Isopropenyl-2-methylcyclopent-1-enecarboxaldehyde	36.11	2.3	711.2013	C_10_H_14_O	34.31	0-00-0
(158)	(-)-Carvone	37.8	2.35	909.8454	C_10_H_14_O	32.76	6485-40-1
(159)	1-Cyclohexene-1-carboxaldehyde, 4-(1-methylethenyl)-	39.85	2.43	1404.104	C_10_H_14_O	31.76	2111-75-3
(160)	3,5-Heptadienal, 2-ethylidene-6-methyl-	36.95	2.28	238.0085	C_10_H_14_O	28.28	99172-18-6
(161)	Benzenemethanol, *α*,*α*,4-trimethyl-	34.05	2.28	288.3869	C_10_H_14_O	16.59	1197-01-9
(162)	Cyclohexanone, 2-(2-butynyl)-	42.38	3.18	59.1028	C_10_H_14_O	15.02	54166-48-2
(163)	D-Verbenone	41.42	2.16	475.2141	C_10_H_14_O	11.63	18309-32-5
(164)	3,5-Heptadienal, 2-ethylidene-6-methyl-	36.11	2.5	184.88	C_10_H_14_O	8.97	99172-18-6
(165)	o-Cymene	23.55	1.55	35991.09	C_10_H_14_	47.51	527-84-4
(166)	2,6-Dimethyl-1,3,5,7-octatetraene, E,E-	31.64	1.71	164.8089	C_10_H_14_	20.19	460-01-5
(167)	Benzene, 1-methyl-4-(1-methylethenyl)-	27.9	1.8	271.6784	C_10_H_12_	20.42	1195-32-0

## References

[1] Shen YJ (2002). *Pharmacology of Traditional Chinese Medicine*.

[2] Gorinstein S, Martín-Belloso O, Park YS (2001). Comparison of some biochemical characteristics of different citrus fruits. *Food Chemistry*.

[3] Bocco A, Cuvelier ME, Richard H, Berset C (1998). Antioxidant activity and phenolic composition of citrus peel and seed extracts. *Journal of Agricultural and Food Chemistry*.

[4] Ma YQ, Ye XQ, Fang ZX, Chen JC, Xu GH, Liu DH (2008). Phenolic compounds and antioxidant activity of extracts from ultrasonic treatment of satsuma mandarin (*Citrus unshiu* Marc.) peels. *Journal of Agricultural and Food Chemistry*.

[5] Ma YQ, Chen JC, Liu DH, Ye XQ (2008). Effect of ultrasonic treatment on the total phenolic and antioxidant activity of extracts from citrus peel. *Journal of Food Science*.

[6] Xu G, Ye X, Chen J, Liu D (2007). Effect of heat treatment on the phenolic compounds and antioxidant capacity of citrus peel extract. *Journal of Agricultural and Food Chemistry*.

[7] Manthey JA, Guthrie N, Grohmann K (2001). Biological properties of citrus flavonoids pertaining to cancer and inflammation. *Current Medicinal Chemistry*.

[8] Choi MY, Chai C, Park JH, Lim J, Lee J, Kwon SW (2011). Effects of storage period and heat treatment on phenolic compound composition in dried *Citrus* peels (Chenpi) and discrimination of Chenpi with different storage periods through targeted metabolomic study using HPLC-DAD analysis. *Journal of Pharmaceutical and Biomedical Analysis*.

[9] Sun Y, Liu Z, Wang J, Wang Y, Zhu L, Li L (2009). Preparative isolation and purification of flavones from Pericarpium Citri Reticulatae by high-speed counter-current chromatography. *Chinese Journal of Chromatography*.

[10] Chizzali E, Nischang I, Ganzera M (2011). Separation of adrenergic amines in *Citrus aurantium* L. var. *amara* by capillary electrochromatography using a novel monolithic stationary phase. *Journal of Separation Science*.

[11] Yin A, Han Z, Shen J (2011). Comparison of essential oil enriched with ultrafiltration method and extraction method respectively from essential oil-in-water emulsion of Citri Reticulatae Pericarpium Viride by GC-MS. *China Journal of Chinese Materia Medica*.

[12] Phillips CA, Gkatzionis K, Laird K (2012). Identification and quantification of the antimicrobial components of a citrus essential oil vapor. *Natural Product Communications*.

[13] Hamdan D, El-Readi MZ, Nibret E (2010). Chemical composition of the essential oils of two Citrus species and their biological activities. *Pharmazie*.

[14] Reichenbach SE, Tian X, Tao Q, Ledford EB, Wu Z, Fiehn O (2011). Informatics for cross-sample analysis with comprehensive two-dimensional gas chromatography and high-resolution mass spectrometry (GC×GC-HRMS). *Talanta*.

[15] Cao G, Shan QY, Li XM (2011). Analysis of fresh *Mentha haplocalyx* volatile components by comprehensive two-dimensional gas chromatography and high - resolution time-of-flight mass spectrometry. *Analyst*.

[16] Marriott P, Shellie R (2002). Principles and applications of comprehensive two-dimensional gas chromatography. *Trends in Analytical Chemistry*.

[17] Shellie R, Marriott PJ (2002). Comprehensive two-dimensional gas chromatography with fast enantioseparation. *Analytical Chemistry*.

[18] Cao G, Cai H, Cong XD (2012). Global detection and analysis of volatile components from sun-dried and sulfur-fumigated herbal medicine by comprehensive two-dimensional gas chromatography/time-of-flight mass spectrometry. *Analyst*.

[19] Qiu Y, Lu X, Pang T, Zhu S, Kong H, Xu G (2007). Study of traditional Chinese medicine volatile oils from different geographical origins by comprehensive two-dimensional gas chromatography-time-of-flight mass spectrometry (GC×GC-TOFMS) in combination with multivariate analysis. *Journal of Pharmaceutical and Biomedical Analysis*.

[20] Ma C, Wang H, Lu X, Li H, Liu B, Xu G (2007). Analysis of *Artemisia annua* L. volatile oil by comprehensive two-dimensional gas chromatography time-of-flight mass spectrometry. *Journal of Chromatography A*.

